# Deaf-1 regulates epithelial cell proliferation and side-branching in the mammary gland

**DOI:** 10.1186/1471-213X-8-94

**Published:** 2008-10-01

**Authors:** Holly E Barker, Gordon K Smyth, James Wettenhall, Teresa A Ward, Mary L Bath, Geoffrey J Lindeman, Jane E Visvader

**Affiliations:** 1 The Walter and Eliza Hall Institute of Medical Research, Parkville, VIC 3050, Australia; 2 Department of Medical Oncology, Royal Melbourne Hospital, Parkville, VIC 3050, Australia

## Abstract

**Background:**

The transcription factor DEAF-1 has been identified as a high affinity binding partner of the LIM-only protein LMO4 that plays important roles in mammary gland development and breast cancer. Here we investigated the influence of DEAF-1 on human and mouse mammary epithelial cells both *in vitro *and *in vivo *and identified a potential target gene.

**Results:**

Overexpression of DEAF-1 in human breast epithelial MCF10A cells enhanced cell proliferation in the mammary acini that develop in 3D cultures. To investigate the effects of Deaf-1 on mammary gland development and oncogenesis, we generated *MMTV-Deaf-1 *transgenic mice. Increased ductal side-branching was observed in young virgin mammary glands, accompanied by augmented cell proliferation. In addition, the ratio of the progesterone receptor isoforms PRA and PRB, previously implicated in regulating ductal side-branching, was altered. Affymetrix gene profiling studies revealed *Rac3 *as a potential target gene and quantitative RT-PCR analysis confirmed that *Rac3 *was upregulated by Deaf-1 in immortalized mouse mammary epithelial cells. Furthermore, *MMTV-Deaf-1 *transgenic mammary glands were found to have elevated levels of *Rac3 *mRNA, suggesting that it is a *bona fide *target.

**Conclusion:**

We have demonstrated that overexpression of *Deaf-1 *enhances the proliferation of human breast epithelial cells *in vitro *and mouse epithelial cells *in vivo*. Transgenic mammary glands overexpressing Deaf-1 exhibited a modest side-branching phenotype, accompanied by an increase in the number of BrdU-positive cells and a decrease in the proportion of PRA-expressing cells. Although proliferation was enhanced in Deaf-1 transgenic mice, overexpression of this gene was not sufficient to induce the formation of mammary tumors. In addition, our studies identified *Rac3*, encoding a small Rho-like GTPase, as a potential target of Deaf-1 in mouse mammary epithelial cells.

## Background

Mammary gland development requires the coordinated action of hormones and growth factors [1]. Embryonic development involves the formation of mammary placodes and rudimentary sprouts, while the majority of mammopoiesis occurs post-natally. Functional development of the mammary gland is initiated at puberty upon secretion of the ovarian hormones estrogen and progesterone which instigate reciprocal signaling between the epithelium and stroma (reviewed in [2]). By 8 – 10 weeks of age in the mouse, complex branching and elongation through the stroma results in an extensive network of ducts filling the entire mammary fat pad. During pregnancy, the secretory alveolar structures develop and functionally differentiate to enable milk production in late pregnancy and milk secretion during lactation. At weaning, the process of involution commences and involves extensive remodeling of the mammary gland to a virgin-like state.

Several signaling pathways and transcription factors have been shown to have essential roles in regulating mammary gland development in the mouse (reviewed in [1,3]). Deformed epidermal autoregulatory factor-1 (DEAF-1) was first isolated in *Drosophila melanogaster *as a novel DNA-binding protein that binds an upstream response element of the homeotic gene *Deformed *[4]. Since its discovery, orthologues in human, rat and monkey have been identified and all exhibit a relatively low degree (46%) of similarity to the *Drosophila *protein [5]. The centrally located SAND domain (Sp100, AIRE-1, NucP41/75 and DEAF-1) along with the MYND domain (myeloid translocation protein 8, Nervy and DEAF-1) in the carboxy-terminus comprise the evolutionarily conserved structural regions of DEAF-1 that have been extensively characterized in other transcription factors. The SAND domain contains a nuclear localization signal (NLS) and appears to confer DNA-binding activity [5]. The MYND domain is a cysteine-rich structure that likely mediates protein-protein interactions [4,5]. DEAF-1 is the only known mammalian protein that contains both a SAND and MYND domain. In *Drosophila*, DEAF-1 plays an important role in embryonic development, particularly in the segmentation stage following cuticle secretion [6].

The mammalian DEAF-1 protein was first identified in an affinity-binding screen using a synthetic retinoic acid response element (RARE). *Deaf-1 *transcripts appear to be widely distributed in rat and mouse tissues [5], with highest levels present in the central nervous system, dorsal root ganglia, submandibular gland, epidermis and mammary placodes of the embryo [7], and the brain, lung and spleen in the adult [5]. At a biochemical level, Deaf-1 has been shown to interact with Lmo4, a LIM-only adaptor protein, as well as with members of the nuclear Clim/Ldb protein family [7]. Disruption of *Deaf-1 *in mice revealed that it is important for neural tube closure and skeletal patterning [8]. *Deaf-1*-deficient mice displayed exencephaly, transformation of cervical segments and rib cage abnormalities, albeit with incomplete penetrance. Interestingly, Lmo4-deficient mice also exhibited neural tube defects and homeotic transformations [8], suggesting that Lmo4 and Deaf-1 act in a complex to mediate specific physiological functions. In the context of mammary tissue, conditional deletion of *Lmo4 *in mouse mammary glands during pregnancy results in impaired alveolar development [9]. Conversely, overexpression of LMO4 has been observed in greater than 50% of human breast cancers and Lmo4 is oncogenic when overexpressed in the mammary glands of transgenic mice [10].

Given that Deaf-1 is expressed in the mammary gland and forms a complex with Lmo4, we explored a potential role for Deaf-1 in mammary epithelial cells. Overexpression of DEAF-1 in MCF10A cells revealed that DEAF-1 plays a role in regulating the proliferation of human breast epithelial cells and increased ductal epithelial proliferation was also observed in young *Deaf-1 *transgenic mice in the post-pubertal phase. Concomitantly, decreased expression of PRA and augmented side-branching were apparent in these mice. In contrast to *Lmo4*, overexpression of *Deaf-1 *in transgenic mice did not induce mammary hyperplasia or tumors. Finally, Affymetrix gene profiling studies were carried out to explore potential Deaf-1 target genes in mammary epithelial cells, leading to the identification of *Rac3 *which encodes a small Rho-like GTPase [11].

## Results

### DEAF-1 enhances the proliferation of human mammary epithelial cells

**Figure 1:**
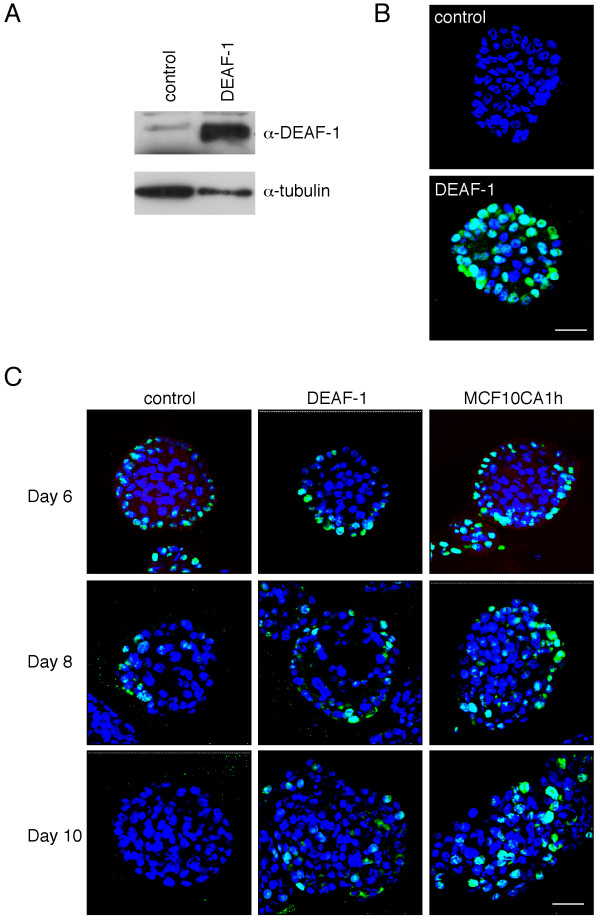
**Overexpression of DEAF-1 in MCF10A cells leads to enhanced cellular proliferation within acini**. A) MCF10A-EcoR cells transduced with either a *Deaf-1*-expressing or empty pBabe-puro retrovirus were analyzed by western blotting of whole cell lysates using anti-DEAF-1 polyclonal antisera. Blotting for tubulin provided a loading control. B) Control or Deaf-1 expressing MCF10-EcoR cells were plated at 4,000 cells/well in eight-well glass chamber slides. Wells were pre-coated with Matrigel and the final culture medium contained 20 ng/ml EGF and 2% Matrigel. Acini were fixed in 2% paraformaldehyde after 8 days in culture, immunostained with anti-DEAF-1 (green), counterstained with DAPI (blue) and acini visualized by confocal microscopy (DAPI = uv; and DEAF-1 = 488 nm). Scale bar represents 47.6 μm. C) Transduced acini were grown as in B) from control MCF10A-EcoR cells, DEAF-1-transduced-MCF10A cells, and malignant MCF10CA1h cells. Cells were fixed in 2% PFA after 6, 8 and 10 days in culture. Acini were immunostained with anti-Ki67 (green) and counterstained with DAPI (blue) following 6, 8 and 10 days in culture. At least three independent experiments were performed. Acini were visualized with a confocal microscope (DAPI = uv; and Ki67 = 488 nm). Scale bar represents 47.6 μm.

To generate human breast epithelial cells stably overexpressing human DEAF-1 protein, MCF10A cells were initially transfected with a vector containing the mouse *Ecotropic Receptor *(*EcoR*) gene to generate MCF10A-EcoR cells. These cells were subsequently infected with an ecotropic pBabe-puro retrovirus encoding DEAF-1. Western blotting of whole cell lysates confirmed that DEAF-1 was overexpressed in *DEAF-1*-transduced MCF10A-EcoR cells relative to control cells transduced with an empty vector (Fig. 1A).

The MCF10A cellular assay described by Debnath et al [12] was used to assess the effect of DEAF-1 on breast epithelial cell proliferation and the formation of acinar structures. Transduced cells were cultured for periods of 6, 8 or 10 days, after which they were immunostained and evaluated by immunofluorescence using confocal microscopy. DEAF-1 expression was detected using an antibody raised against an amino-terminal peptide in the DEAF-1 protein. Acini generated from MCF10A-EcoR cells transduced with the DEAF-1 retrovirus expressed high levels of DEAF-1 protein, whereas DEAF-1 expression was undetectable in control acini (Fig. 1B). To assess cell proliferation, acini were immunostained with anti-Ki67 antibody. In general, proliferating cells were found to be restricted to the periphery of acini and were evident in both control and DEAF-1-expressing acini following 6 and 8 days of culture. By 10 days, proliferation ceased in control acini which appeared organized and spherical (Fig. 1C, lower left panel). In contrast, DEAF-1 overexpressing acini continued to proliferate, with a commensurate increase in the size and number of acini (Fig. 1C, lower middle panel). The malignant isogenic MCF10CA1h cell line was included as a control as it generates abnormal acinar structures. The MCF10CA1h cell line was derived from tumors arising in mice that had been injected with the HA-ras transformed MCF10AneoT cells, following transduction of parental MCF10A cells [13,14]. As expected, proliferation in MCF10CA1h acini was prolonged (Fig. 1C, lower right panel) and Ki67-positive cells were observed up to 21 days in culture (data not shown). Reminiscent of MCF10CA1h acini, DEAF-1 overexpressing acini also appeared more disorganized. Apoptosis and polarity were assessed by TUNEL and anti-GM130 staining respectively but no differences were observed (data not shown).

### Generation of Deaf-1 transgenic mice

**Figure 2:**
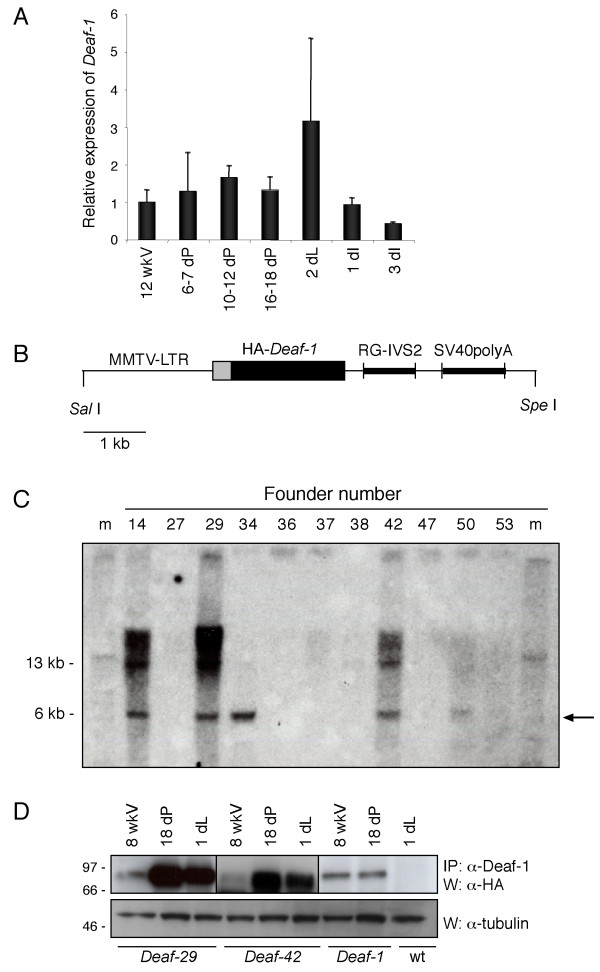
**Generation and analysis of Deaf-1 transgenic mice**. A) Quantitative RT-PCR analysis of RNA purified from mouse mammary glands at different stages of development (wkV, week-old virgin; dP, days pregnant; dL, days lactation; and dI, days involution) using *Deaf-1 *and *CK18 *specific primers. Data are from at least two wild-type mice for each time-point and error bars represent standard deviation from the mean. B) Schematic diagram of the 7 kb transgenic construct showing the *MMTV *promoter, the HA-*Deaf-1 *transgene, and the rabbit β-globin plus SV40poly(A) intronic sequences. C) Southern blot analysis of HindIII-digested tail DNA, probed with a [α-^32^P]-labeled SV40 fragment. Arrow indicates the *Deaf-1 *fragment. D) Western blot analysis of protein lysates from 8 week-old virgin (8 wkV), 18 days pregnant (18 dP) and 1 day lactating (1 dL) mammary glands from *Deaf-29*, *Deaf-42 *and *Deaf-1 *transgenic strains. Anti-Deaf-1 and anti-HA antibodies were used to detect the HA-tagged Deaf-1 protein. Wild-type mammary gland lysate provided a negative control (upper panel). Samples of each lysate (prior to immunoprecipitation) were subject to immunoblotting with anti-tubulin to control for protein loading (lower panel).

To evaluate the expression of *Deaf-1 *during mammary development, RNA was isolated from mouse mammary glands at different developmental stages and subjected to quantitative real-time PCR analysis. Expression of *Deaf-1 *was quantified relative to that of the luminal epithelial marker cytokeratin 18 (*CK18*) and was found to be expressed at all stages of mammary gland development, with slightly higher expression observed during pregnancy and lactation (Fig. 2A).

To investigate the effects of Deaf-1 overexpression on mammary gland development and oncogenesis, we generated transgenic mice expressing a HA-tagged mouse *Deaf-1 *(full-length) gene under the control of the mouse mammary tumor virus-long terminal repeat (MMTV-LTR). The MMTV-LTR is active in both virgin and pregnant mammary glands [15] but reaches maximal activity during pregnancy since the long terminal repeat (LTR) of the *MMTV *promoter is activated by steroid hormones. The transgene included rabbit β-globin and simian virus 40 (SV40) intronic sequences to augment mRNA stability, as well as a polyadenylation (poly(A)) sequence (Fig. 2B). Southern blot analysis of genomic DNA from the offspring of founder mice demonstrated that five lines transmitted the transgene with variation in copy number evident (Fig. 2C). Expression of the transgene was assessed by immunoprecipitation of mammary protein lysates from virgin, pregnant (18 dP) and lactating (1 dL) mice with anti-Deaf-1 polyclonal antisera, followed by Western blot analysis using an anti-HA antibody. Abundant expression of the transgene was observed during pregnancy and lactation in two strains, *Deaf-29 *and *Deaf-42 *(Fig. 2D, upper panel). Immunoblotting of lysates prior to immunoprecipitation with anti-tubulin antibody provided a loading control (Fig. 2D, lower panel).

### *Deaf-1 *transgenic mammary glands exhibit increased side-branching in young mice

**Figure 3:**
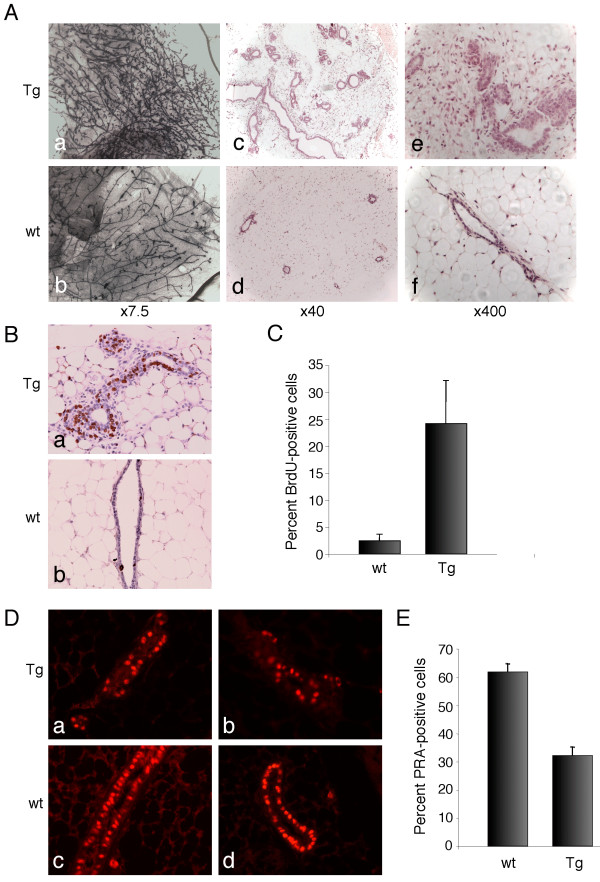
**Increased side-branching and proliferation in the mammary glands of young Deaf-1 transgenic mice**. A) Wholemounts of thoracic mammary glands harvested from transgenic (a) and wild-type (b) 8 week-old virgin mice. Original magnification, *×7.5*. Sections of inguinal mammary glands from transgenic (c and e) and wild-type (d and f) mice stained with haematoxylin and eosin. Original magnification, *×40 *(c and d) and *×400 *(e and f). B) Immunostaining of mammary sections from transgenic (a) and wild-type (b) 8 week-old mice with anti-BrdU. Original magnification *×400*. C) Percent BrdU-positive cells was calculated by counting greater than 1,000 nuclei in 10 random fields for each mouse. At least 5 mice were analyzed for both transgenic and wild-type genotypes. The percentage of BrdU-positive cells in transgenic glands (Tg) was 24.2% ± 7.9% versus 2.5% ± 1.2% in control (wt) glands. Error bars represent standard deviation from the mean. D) Immunostaining of sections from transgenic (a and b) and wild-type (c and d) mammary glands of 8 week-old mice using anti-PRA antibody. Original magnification *×400*. E) Percent PRA-positive cells was calculated by counting greater than 1,000 nuclei in 10 random fields for each mouse. At least 4 mice were analyzed for each genotype. The percentage of PRA-positive cells in transgenic glands (Tg) was 32.1% ± 3.0% versus 61.8% ± 2.9% in control (wt) glands. Error bars represent the standard deviation from the mean.

Analysis of young virgin transgenic mice (8 weeks of age) revealed an increase in the number of side-branches by wholemount and histological analyses (Fig. 3A). Since fluctuation in ductal branching occurs during the estrous cycle, vaginal smears were stained with haematoxylin and eosin to ensure that mammary glands were harvested from mice at the same stage of the estrous cycle. For strain *Deaf-29*, 5 out of 12 mice exhibited mammary glands with substantially increased side-branching compared with 2 out of 5 *Deaf-42 *transgenic mice (Table 1). Although some variation in the degree of side-branching was noted amongst the sixteen control (wild-type) mice analyzed, transgenic mice exhibited profoundly abnormal mammary glands compared to wild-type glands.

**Table 1:** Number of *Deaf-29 *and *Deaf-42 *transgenic mice exhibiting a side-branching phenotype

**Transgenic Line**	Phenotype/Total*
Deaf-29	5/12
Deaf-42	2/5

* Number of mice at 8 weeks exhibiting a phenotype/total number of transgenic mice analyzed.

### Increased proliferation and altered expression of PRA and PRB in *Deaf-1 *transgenic glands

Side-branching occurs concomitantly with epithelial cell proliferation in pubertal mammary glands. *In vivo *BrdU labeling was therefore used to quantify proliferation in transgenic and wild-type glands. Transgenic 8 week-old virgin mice showed a marked increase in the number of proliferating cells (24.2 ± 7.9% BrdU-positive ductal epithelial cells in transgenic glands compared to 2.5 ± 1.2% in wild-type glands, Figs. 3B and 3C). TUNEL staining was employed to quantify the number of apoptotic cells in the virgin glands but few TUNEL-positive cells were apparent and no difference was observed between transgenic and wild-type glands (data not shown).

The progesterone receptor (PR) isoforms together with Wnt4 play important roles in development of the mammary ductal tree during puberty [16-18]. To assess the expression of PR in virgin mammary glands of transgenic mice, immunofluorescence was performed using anti-PRA and anti-PRB specific antibodies. Fewer ductal epithelial cells were positive for PRA in transgenic mammary glands relative to those from wild-type mice (Fig. 3D). Detection of the PRB isoform by immunostaining proved difficult, although low levels were seen in transgenic and wild-type glands (data not shown). To quantify the number of PRA-positive cells in wild-type versus transgenic mammary glands, immunohistochemistry was performed: 32.1 ± 3.0% ductal epithelial cells were PRA-positive in transgenic glands compared to 61.8 ± 2.9% in wild-type glands (Fig. 3E). Wnt4 expression was assessed by semi-quantitative RT-PCR analysis but no difference was observed between transgenic and wild-type glands (data not shown).

### *Deaf-1 *transgenic mammary glands appear normal during pregnancy, lactation and involution and do not develop tumors

**Figure 4:**
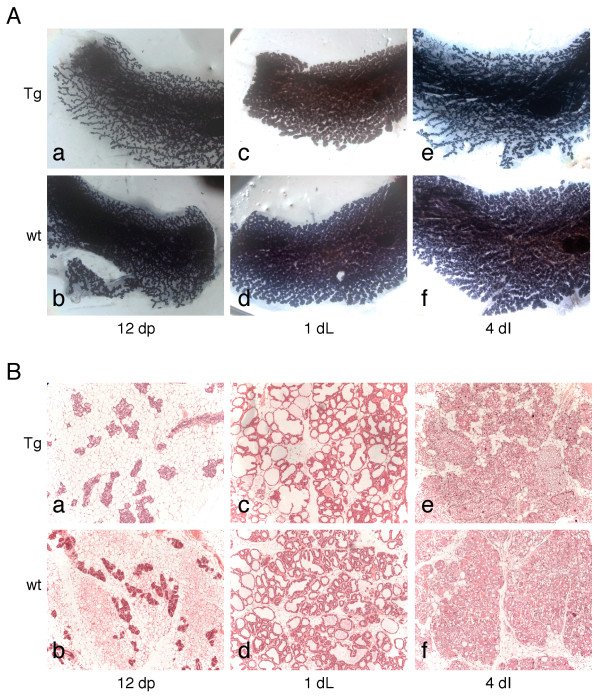
**No phenotype is evident in Deaf-1 transgenic mice during pregnancy, lactation and involution**. A) Wholemounts of inguinal mammary glands from *Deaf-1 *transgenic (a, c and e) or FVB/N wild-type mice (b, d and f). Whole-mounted glands at different time-points: 12 days of pregnancy (12 dP; a and b), 1 day of lactation (1 dL; c and d) and 4 days of involution (4 dI; e and f). Original magnification *×7.5*. B) Sections of glands from *Deaf-1 *transgenic (a, c and e) and FVB/N wild-type mice (b, d and f) at 12 days of pregnancy (12 dP; a and b), 1 day of lactation (1 dL; c and d) and 4 days of involution (4 dI; e and f). Original magnification *×100*. Tg, transgenic; wt, wild-type.

Wholemounts and histological sections were prepared from transgenic (*Deaf-29 *and *Deaf-42*) and wild-type mice at the following stages of mammary gland development: 12 and 18 days of pregnancy, 1 and 8 days of lactation, and 1 and 4 days of involution. All transgenic mammary glands appeared morphologically normal. Figure 4 shows representative wholemounts and sections harvested from transgenic and wild-type mice at pregnancy, lactation and involution. At least three mice of each genotype were analyzed for each stage.

To investigate the oncogenic potential of Deaf-1 in the mammary gland, pituitary isografting was employed to recapitulate pregnancy and increase MMTV-LTR-driven *Deaf-1 *expression [19]. Two pituitary glands from FVB/N mice were transplanted into each inguinal mammary gland of 7 week-old transgenic and wild-type mice, which were then maintained for 12 months. Mice presenting with tumors (or another illness) prior to 12 months were sacrificed early. Although two out of thirteen (15.4%) *Deaf-29 *transgenic mice developed mammary tumors within 12 months of age, one out of ten wild-type mice (10%) developed a tumor during this period. Moreover, Deaf-1 transgenic glands did not exhibit hyperplasia. We therefore conclude that overexpression of Deaf-1 is not sufficient to induce mammary tumors.

### *Rac3 *is a potential target gene of Deaf-1 in the mammary gland

**Figure 5:**
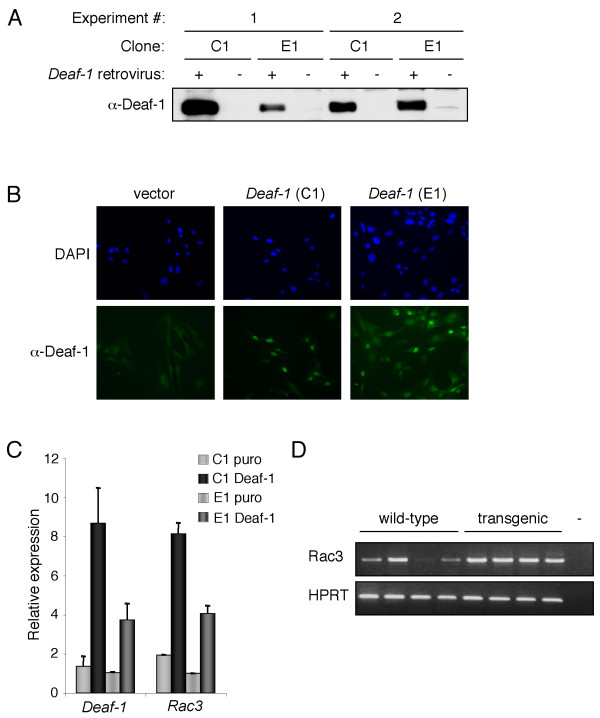
***Rac3 *is a candidate Deaf-1 target gene**. Expression of Deaf-1 in whole cell lysates from cells infected with a *Deaf-1 *retrovirus (+) or control retrovirus (-). Two independent clones (C1 and E1) were generated and used for these studies. Western blots were probed with anti-Deaf-1 antibody. B) Clones transduced with either a *Deaf-1 *or control retrovirus were plated on coverslips and immunostained with anti-Deaf-1 polyclonal antisera, followed by FITC-conjugated anti-rabbit antibody. Cells were counterstained with DAPI to visualize nuclei by fluorescence microscopy (clones C1 and E1 are shown). C) Quantitative RT-PCR analysis was performed on cDNA generated from Affymetrix RNA samples using primers specific for *Deaf-1*, *Rac3 *and *18S rRNA*. Data are from two independent experiments and error bars represent the standard deviation from the mean. D) Semi-quantitative RT-PCR analysis of RNA prepared from wild-type or Deaf-1 transgenic mammary glands of 8 week-old mice using primers specific for *Rac3 *and *HPRT*.

To investigate potential target genes of Deaf-1, primary mammary epithelial cells (MECs) were isolated from a *Deaf-1*^-/- ^mouse and immortalised by transduction with a retrovirus encoding the human papilloma virus (HPV16) E6/E7 proteins. Two MEC clones were selected and transduced with either an empty retrovirus or one encoding *Deaf-1*. RNA was subsequently isolated from cells at 24 and 48 hours post-transduction for Affymetrix analysis and protein lysates were prepared for Western blot analysis. In addition, cells were plated on coverslips to assess Deaf-1 protein expression at each time-point by immunofluorescence. High levels of Deaf-1 expression were demonstrated in cells transduced with the *Deaf-1 *retrovirus relative to control cells following a 48 hour selection in puromycin (Figs. 5A and 5B). This time-point was therefore selected for further analysis.

To compare the gene expression profiles of *Deaf-1*-transduced MECs relative to *Deaf-1*-deficient MECs, Affymetrix analysis was performed using the GeneChip^® ^Mouse Expression Set 430 2.0 array which comprises 39,000 transcripts on a single array. Total RNA was harvested from two independent clones (C1 and E1) infected with either control or Deaf-1-expressing retrovirus, representing four samples in total. Clones C1 and E1 were treated as biological replicates in the subsequent analysis. Notably, the top gene differentially expressed between Deaf-1 and control samples following hybridization to Affymetrix chips was *Deaf-1 *itself. Table 2 lists the top 10 genes that were expressed in a differential manner with a p-value less than 0.01. This order was based on calculation of the t statistic, which is a measure of consistency between the two clones, C1 and E1. The top five differentially expressed genes obtained from this analysis were: *Deaf-1*, *Eif4g3*, *Agbl5*,*Kng1 *and *Rac3*.

**Table 2:** Top 10 differentially expressed genes between Deaf-1 and control samples

ID	Name	M	A	t	p-value
1448446_at	Deaf1	1.529607	5.723798	8.519405	0.000124
1426833_at	Eif4g3	1.086468	6.67216	6.415899	0.000609
1439465_x_at	Agbl5	0.907762	5.675668	4.825747	0.00272
1416676_at	Kng1	0.826599	3.34331	4.609687	0.003417
1420554_a_at	Rac3	0.726538	5.085094	4.440323	0.004105
1431806_at	4931408D14Rik	0.737322	3.439207	4.37871	0.004393
1433512_at	Fli1	-0.93092	3.370952	-4.29734	0.004808
1441639_at	Zcchc8	0.822497	3.174238	4.291322	0.004841
1444199_at	unannotated	0.680436	4.035815	4.182175	0.005473
1434239_at	Rrp12	-0.66472	5.164167	-4.084	0.006122

*Rac3 *appeared to be of most interest since its expression has previously been reported in breast cancer epithelial cells [11]. To validate whether *Rac3 *was a potential target of Deaf-1, real-time RT-PCR analysis was performed. An increase in *Rac3 *levels was confirmed (Fig. 5C), as was also observed by semi-quantitative RT-PCR analysis (data not shown). To further examine the correlation between *Rac3 *and Deaf-1 expression, RT-PCR analysis was performed on RNA derived from wild-type and *Deaf-1 *transgenic mammary glands. Increased expression of *Rac3 *was observed in *MMTV-Deaf-1 *transgenic samples (Figure 5D). Although a single wild-type mouse showed the same level of *Rac3 *mRNA as transgenic glands, all other wild-type mice exhibited substantially lower levels of *Rac3*. Therefore, it seems likely that *Rac3 *represents a bona fide target gene.

## Discussion

The Deaf-1 transcription factor has been implicated in a number of developmental processes, including skeletal patterning and neural tube closure in the mouse embryo [8]. To further understand the role of Deaf-1 in breast epithelial cells, we examined the effect of DEAF-1 overexpression in MCF10A cells and in the mammary glands of transgenic mice. MCF10A breast epithelial cells exhibit many characteristics of normal breast epithelium including hormone and growth factor-responsive growth in three-dimensional cultures and the inability to grow in an anchorage-independent manner [20]. Overexpression of DEAF-1 in MCF10A cells was found to enhance cellular proliferation in mammary acini but no change was observed in the number of apoptotic cells or in cell polarity.

Overexpression of *Deaf-1 *in the mammary glands of transgenic mice led to a proliferative defect and a striking increase in the number of side-branches in young post-pubertal mice. The phenotype observed in the *Deaf-1 *transgenic mice was transient as it was no longer evident in older adult mice at 12 weeks of age. No pronounced defects were evident in *Deaf-1*-deficient mice, possibly reflecting compensatory mechanisms (unpubl. data). Interestingly, a transient delay in ductal development has previously been observed in virgin mice expressing a dominant negative form of Lmo4 (engrailed-Lmo4 fusion protein) [21]. A transient phenotype has also been reported in Bim knockout mice [22]. The increased number of side-branches occurring within the mammary glands of young females in two transgenic strains was accompanied by a significant increase in the proportion of BrdU-positive cells. In addition, decreased expression of the progesterone receptor isoform PRA was observed in *MMTV-Deaf-1 *transgenic mammary glands. Approximately 62% of ductal epithelial cells in wild-type mammary glands at 8 weeks expressed PRA, in agreement with previous findings [23,24]. However, a two-fold decrease in the number of PRA-positive ductal epithelial cells was apparent in transgenic mammary glands. In the mouse mammary gland it is relevant that the progesterone receptor exists predominantly as the PRA isoform, and that the ratio of PRA to PRB is estimated to be 2:1 [25]. It has previously been shown that PRB is essential for tertiary side-branching in the mammary gland during puberty [26] and that PRA has the ability to suppress PRB-mediated mammary proliferation [27]. Interestingly, the majority of PRA-expressing epithelial cells do not proliferate in the virgin mammary gland, whereas PRB colocalises extensively with BrdU-labeled cells, a marker of proliferation [18,23]. Thus the increased proliferation and aberrant side-branching observed in *Deaf-1 *transgenic mice may reflect an elevated PRB:PRA ratio, resulting from decreased expression of PRA in these glands. The *PRA *gene could not be identified as a target in the Affymetrix analysis since a specific probe for *PRA *does not exist. It therefore remains to be determined whether down-regulation of *PRA *expression by Deaf-1 occurs via a direct or indirect mechanism.

Interrogation of Affymetrix gene arrays revealed a small number of genes that were differentially expressed in Deaf-1-expressing MECs versus Deaf-1-null MECS. Only the top two genes exhibited a greater than two-fold change but all genes in Table 2 had high t statistic values. Elevated *Rac3 *levels in Deaf-1-expressing MECs could be demonstrated by real-time PCR analysis. Functional analysis of the promoter region of *Rac3 *revealed the presence of multiple Deaf-1 binding sites but luciferase reporter assays were inconclusive (data not shown). This does not preclude direct regulation of *Rac3 *by Deaf-1 via further upstream or downstream regulatory elements and requires more extensive analyses using a large genomic region spanning the entire *Rac3 *gene. Interestingly, mammary glands of *Deaf-1 *transgenic mice had increased levels of *Rac3 *mRNA compared to wild-type mammary glands. Thus, it appears that *Rac3 *may be a genuine target gene of Deaf-1. Recently, a study of transgenic mice expressing activated *Rac3 *in the mammary epithelium revealed that the glands underwent incomplete involution, with epithelial islands persisting up to nine months postpartum. These mice also developed benign mammary gland lesions [28]. We did not observe delayed involution in *Deaf-1 *transgenic mice analyzed. However, mice were only analyzed up to 12 days postpartum (data not shown). Further assessment of the relationship between Rac3 and Deaf-1 may require analysis of *Deaf-1 *transgenic mammary glands several months postpartum to determine whether Deaf-1 overexpression leads to altered mammary gland physiology.

Activated Rac3 has been linked to deregulated p21-activated kinase (Pak) and c-Jun N-terminal kinase (JNK) activities in human cancer cells [29]. Subsequently, activation of Rac3 was found to be critical for integrin and growth factor-mediated regulation of cellular migration and adhesion [30], which are important steps in the progression of metastatic disease. Depletion of Rac3 from BT549 breast carcinoma cells by RNAi strongly inhibited cell invasion revealing a role for this GTPase in breast cancer metastasis [31]. Intriguingly, these functions may parallel the proliferative, migratory and invasive functions ascribed to LMO4, a partner of DEAF-1, in human breast cancer cell lines [10]. Although *Lmo4 *transgenic mice develop hyperplasia and mammary intraepithelial neoplasia or adenosquamous carcinoma [10], overexpression of *Deaf-1 *did not lead to mammary tumors. Further insight may come from investigating *Rac3 *as a potential target gene of the endogenous DEAF-1/LMO4 protein complex in normal and cancerous breast epithelial cells.

## Conclusion

Our data have revealed that the transcription factor DEAF-1 regulates the proliferation of human and mouse mammary epithelial cells. Enforced expression of *Deaf-1 *in transgenic mice led to increased proliferation and side-branching in post-pubertal mammary glands but was not oncogenic. Together with the perturbed PRA:PRB ratio and the known role of PR in side-branching, these data suggest that Deaf-1 may regulate the expression of PR. Finally, we have identified the small GTPase-encoding gene *Rac3 *as a potential target of Deaf-1 in the developing mammary gland.

## Methods

### Three-dimensional MCF10A assay

Stably transfected MCF10A-EcoR cells were passaged, and subjected to 3D assays in GFR Matrigel and indirect immunofluorescence as previously described [12]. Acini were incubated with primary antibodies (anti-Deaf-1 polyclonal antisera; diluted 1:50, anti-Ki67; diluted 1:300; Novo Castra) overnight at 4°C. Secondary antibody (Alexa Fluor^®^-conjugated anti-rabbit-488; Molecular Probes, Invitrogen, Carlsbad, CA, USA) was diluted 1:200. Nuclei were counterstained with 4',6-diamidino-2-phenylindole (DAPI; Sigma, St Louis, MO, USA).

### Generation and analysis of *Deaf-1 *transgenic mice

A fragment encompassing an HA-tagged *Deaf-*1 cDNA was cloned downstream of a MMTV-LTR vector which also contained the simian virus 40 (SV40) untranslated (UTR) and poly(A) sequences [10]. The *MMTV-HA-Deaf-1 *plasmid was digested with *SalI *and *SpeI *to release the *MMTV-HA-Deaf-1-SV40 *fragment from the vector, and subsequently isolated by gel electrophoresis in a 0.7% low melting point agarose gel. The 7 kb fragment was microinjected into the pronucleus of FVB/N fertilised eggs, and these were then transferred to the oviducts of foster mothers (FVB/N). Genomic (tail) DNA from the founder mice was genotyped by PCR. Eleven founder mice were positive for the transgene and mated with wild-type FVB/N mice to obtain F1 generations. All animal experiments were conducted according to the WEHI Animal Ethics Committee guidelines.

Mammary glands were collected from adult female mice at different developmental time-points. Timed pregnancies were scored by observation of vaginal plugs, and confirmed by examination of embryos at the time of mammary gland collection. Lactation time-points corresponded to days post-parturition. Pups were removed after 10 days to initiate involution. The estrous cycle stage of virgin females was determined by vaginal smears, which were dried and stained with haematoxylin and eosin.

### Southern blotting

Genomic tail DNA from founder mice was digested with *HindIII*, separated by electrophoresis, transferred to Hybond N^+ ^membranes (Amersham Biosciences, Buckinghamshire, England) and then hybridized to a 1.2 kb SV40 fragment labeled with [α-^32^P]-dCTP, using the DECAprime™II random primed DNA labeling kit (Ambion, Austin, TX, USA), according to the manufacturer's instructions.

### Histological sections and mammary gland wholemounts

For histological sections, portions of the inguinal mammary gland were harvested and fixed overnight in 4% (w/v) PFA in PBS, pH 7.4, at 4°C. Mammary glands were then embedded in paraffin, and 1.5 μm sections stained with haematoxylin and eosin. For wholemount analysis, whole thoracic and inguinal mammary glands were fixed overnight in Carnoy's solution (60% ethanol, 30% chloroform, 10% acetic acid) before staining with haematoxylin.

### Lysate preparation, immunoprecipitation and Western blot analysis

Protein lysates from adult mouse organs and mammary epithelial cell cultures were prepared in KALB lysis buffer (150 mM NaCl; 1 mM EDTA; 50 mM Tris.HCl, pH 7.5;10 mM NaF; 1 mM Na_3 _VO_4_; 1% Triton X-100)containing Complete protease inhibitor cocktail (Roche, Mannheim, Germany). For immunoprecipitation, 2 μl of anti-Deaf-1 polyclonal antisera was added to 500 μg of protein in a final volume of 200 μl KALB lysis buffer. Samples were incubated on ice for 2 hours before incubation with protein G sepharose beads, washing and Western blot analysis.

Tissue protein lysates (30 – 40 μg of total protein), whole cell lysates (10 – 25 μg of total protein) or immunoprecipitates were resolved on 4–20% Tris-glycine Novex pre-cast polyacrylamide gels (Invitrogen, Carlsbad, CA, USA) and transferred to Immobilon-P polyvinylidene difluoride membranes (PVDF; Millipore, Bedford, MA, USA) using the Novex transfer apparatus. Membranes were then probed with the following primary antibodies: anti-Deaf-1 rabbit polyclonal antisera which was raised against a 16-mer KLH-conjugated peptide (MEDSDSAAKQLQLAEC) located in the amino-terminal of murine Deaf-1 (also recognizes human DEAF-1) diluted 1:1,000, anti-HA rat monoclonal (3F10; Roche) diluted 1:750, and anti-tubulin mouse monoclonal (B-5-1-2; Sigma) diluted 1:5,000.

### Bromodeoxyuridine (BrdU) immunodetection

Mice were injected with 0.5 mg/10 g body weight 5-bromodeoxyuridine (BrdU) Cell Labeling Reagent (Amersham Biosciences, Buckinghamshire, England) 1 hr prior to tissue collection. For immunohistochemical detection of BrdU-labeled cells in mouse mammary glands, sections were treated with 3% hydrogen peroxide and permeabilized with 20 μg/ml proteinase K (Sigma, St Louis, MO, USA) followed by 0.2% Triton X-100. They were then incubated sequentially with rat anti-BrdU antibody (BD Biosciences, Bedford, MA, USA), biotinylated rabbit anti-rat IgG secondary antibody (Dako Cytomation, Carpinteria, CA, USA), and HRP-conjugated strepdavidin (LSAB2; Dako Cytomation), before staining with 3,3'-diaminobenzidine (DAB; Dako Cytomation) and counterstaining with haematoxylin. For quantification of proliferating cells within the mammary glands of transgenic and wild-type mice, greater than 1,000 epithelial nuclei in 10 random fields (×400 magnification) were counted.

### Immunohistochemistry of mouse mammary sections

Sections were deparaffinized, rehydrated and subjected to antigen retrieval by boiling in 10 mM citrate buffer, pH 6.0 for 20 min, before blocking in 10% normal goat serum (NGS). The primary antibodies, anti-PRa6 (anti-PRB) and anti-PRa7 (anti-PRA), kind gifts from C. Clarke and D. Graham, were diluted 1:40 and 1:80 respectively and incubated overnight at 4°C. For immunofluorescence, sections were incubated with anti-mouse-Alexa Flour^®^-594 (Molecular Probes, Invitrogen, Carlsbad, CA, USA), mounted with Fluorescent Mounting Medium (Dako Cytomation, Carpinteria, CA) and visualized by fluorescence microscopy. For immunohistochemistry, sections were incubated with biotinylated anti-mouse IgG secondary antibody (Vector Laboratories Inc, Burlingame, CA, USA) diluted 1:500. The tertiary step, counterstaining and quantification were carried out as described above.

### Generation of mouse mammary epithelial cells and immunofluorescence

Mammary glands were harvested from 8 week-old female *Deaf-1*^-/- ^mice and digested to obtain primary mammary epithelial cells (MECs). MECs were immortalized with an ecotropic retrovirus encoding human papilloma virus (HPV16) E6/E7 proteins as described in [32].

To analyze Deaf-1 expression, cells were plated on coverslips at 0.5 × 10^6 ^cells/plate in 6-well plates, infected with control or *Deaf-1 *retrovirus and then selected in puromycin (1.5 μg/ml) for 48 – 72 hr. Cells were incubated with anti-Deaf-1 rabbit polyclonal antibody before incubation with the anti-rabbit-FITC secondary antibody. Nuclei were counterstained with 4',6-Diamidino-2-phenylindole (DAPI) and visualized by fluorescence microscopy.

### Affymetrix analysis

RNA was purified using the QIAGEN RNeasy Kit (Qiagen, Hilden, Germany) and quality assessed by spectrophotometry and gel electrophoresis before hybridization to Affymetrix slides (GeneChip^® ^Mouse Expression Set 430 2.0). Quality assessment for Affymetrix arrays is reviewed in [33] and was carried out in the Bioinformatics Department (WEHI, Melbourne, Australia). Gene symbols were obtained from the Affymetrix probe-set annotation file, version 25, 19 March 2008.

### RT-PCR and Quantitative RT- PCR analysis

PCR was performed using 1 μl of cDNA and 50 ng of forward and reverse primers (Sigma-Genosys, Sydney, Australia; see Table 3). PCR conditions were as follows: 94°C for 2 min, followed by 30–35 cycles of denaturation at 94°C for 45 sec, annealing at x°C (see Table 3) for 45 sec and extension at 72°C for 45 sec, followed by a final extension at 72°C for 5 min.

**Table 3:** Primers for RT-PCR and quantitative real-time-PCR analysis

**RT-PCR**			
**Transcript**	**Sequence (5'-3')**	**Annealing Temp (x°C)**	**Amplicon size (bp)**

***Rac3***			
forward	GATGGTGGATGGGAAGCCAGTTAAC	68	300
reverse	GGATGGCCTCGTCGAACACTGTC		
***HPRT***			
forward	CACAGGACTAGAACACCTGC	65 (+ 5% glycerol)	229
reverse	GCTGGTGAAAAGGACCTCT		
			

**Quantitative real-time PCR**			

**Transcript**	**Sequence (5'-3')**		**Amplicon size (bp)**

***Deaf-1***			
forward	AGAATGAGCTGCCCACAACT		133
reverse	TCAAAGGTCAGTGCTCCAGA		
***Rac3***			
forward	CACACACACCCATCCTTCTG		100
reverse	TAGGTTATGGGTGCCAGCTT		
***18S rRNA***			
forward	GTAACCCGTTGAACCCCATT		152
reverse	CCATCCAATCGGTAGTAGCG		
***CK18***			
forward	CAAGATCATCGAAGACCTGAGGC		384
reverse	TGTTCATACTGGGCACGGATGTCC		

Quantitative RT-PCR assays were performed in a Rotor-Gene™ 6000 (Corbett Research, Mortlake, NSW, Australia) using 2 μl of cDNA in a 20 μl reaction volume containing 50 ng of each primer (Table 3) and 1 × SensiMix *Plus *SYBR^®^Green I Master reaction mix (Quantace Ltd, London, UK). The amplification program included an initial denaturation step at 95°C for 10 min, followed by 40 cycles of denaturation at 94°C for 15 sec, annealing at 60°C for 30 sec, and extension at 72°C for 30 sec. The *18S rRNA *and *CK18 *analyses were used for normalization to enable construction of standard curves. Relative concentrations were calculated using the delta CT method on the Rotor-Gene™ 6000 software.

## Availability and requirements



## Authors' contributions

HEB designed some of the experiments, performed most of the experimental work and wrote the first draft of the manuscript. GKS and JW carried out the statistical analysis of the Affymetrix data. TAW carried out the real-time analyses and PCR experiments. MLB generated the transgenic mice. JEV and GJL conceived and designed the study and contributed to the writing. All authors reviewed and approved the manuscript.
